# 

**Published:** 1852-01

**Authors:** 


### CONTENTS:

### PART I.—ORIGINAL COMMUNICATIONS.

ART. 1.—On the Therapeutical employment of Collodion in Erysipelas, Articular Rheumatism, Puerperal Peritonitis, etc. By M. R. Latour, - - - - 307
ART. 2.—Notes of a Rattle-Snake Bite admitted into the Ill. Gen. Hospital. By John E. McGirr, M. D., - - - - - - - - - 311
ART. 3.—On the uses of Tobacco in Inflammatory Diseases. By W. Matthews, M. D., 315
" 4.—Cerebro Arachnitis. By J. B. Nash, M. D., - - - - - - 319

### PART II.—REVIEWS AND NOTICES OF NEW WORKS.

ART. 1.—Elements of General ond Pathological Anatomy; presenting a view of the present state of Knowledge in these Branches of Science. By D. Craigie, M. D., 322
ART. 2.—Surgical Anatomy. By Jos Meclaise, - - - - - - 323
" 3—Ranking's Half Yearly Abstract of the Medical Sciences for July, - - 324
" 4.—Elements of Physiology. By W. B. Carpenter, M. D., - - - - 324
" 5.—The Pharmacopœia in the United States of America, - - - 325
" 6.—The Laws of Health, in relation to the Mind and Body. By L. J. Beale, - 325
" 7.—Urinary Deposites; their Diagnosis, Pathology, etc. By G. Bird, A. M., etc., 330
" 8.—The Microscopist, or a Complete Manual on the use of the Microscope, - 331
" 9.—Hints to the People upon the Profession of Medicine. By W. M. Wood, M. D. 332
" 10.—Operative Surgery. By J. F. Malgaigne. - - - - - - 332
" 11.—The Pocket Formulary and Synopsis of the British and Foreign Pharmacopœis: By Henry Beasley, - - - - - - - - - 336
" 12.—Intermarriage or the mode in which, and the causes why, Beauty, Health and Intellect, result from certain unions, and Deformity, Insanity and Disease from others. By Alexander Walker, - - - - - - - 337
" 13.—The Physician's Visiting List, Diary and Book of Engagements for 1852, - 339
" 14.—Essays on Infant Therapeutics. By John B. Beck, M. D., - - - - 340

### PART III.—SELECTIONS.

ART. 1.—Paralysis of the Muscles of Deglutition. By Alfred C. Post, M. D., - 341
" 2.—Case of Aneurism of the Aorta simulating Phthisis. By W. B. McCready, M. D. 343
" 3.—Prevalence of Quackery in America. By James H. Stuart, A. M., M. D., 345
" 4.—On Ovarian Irritation. By F. Churchill, M. D., - - - - - 350
" 5.—On the Protective Power of Vaccination. By S. Annan, M. D., - - 356
" 6—Diet in Protracted Fevers. By E. B. Haskins, M. D., - - - 360
" 7—Kineispathy, - - - - - - - - - - - 263
" 8.—Practical Items, - - - - - - - - - - 366

### PART IV.—EDITORIAL.

ART. 1.—Indiana Hospital for the Insane, - - - - - - - 371
" 2.—Free Medical Schools, - - - - - - - - - 378
" 3.—Mrscellaneous Medical Intelligence, - - - - - - 379
Rush Medical College—Obituary, - - - - - - - 380
New Arrangement, - - - - - - - - - 381

